# CD13/Aminopeptidase N overexpression by basic fibroblast growth factor mediates enhanced invasiveness of 1F6 human melanoma cells

**DOI:** 10.1038/sj.bjc.6603157

**Published:** 2006-05-09

**Authors:** D Fontijn, M C A Duyndam, M P A van Berkel, Y Yuana, L H Shapiro, H M Pinedo, H J Broxterman, E Boven

**Affiliations:** 1 Department of Medical Oncology, VU University Medical Center, De Boelelaan 1117, 1081 HV Amsterdam, The Netherlands; 2 Department of Cell Biology, University of Connecticut, Farmington, CT, USA

**Keywords:** bFGF, CD13, melanoma, invasion

## Abstract

CD13/Aminopeptidase N (CD13) is known to play an important role in tumour cell invasion. We examined whether basic fibroblast growth factor (bFGF) is involved in the regulation of CD13 expression in human melanoma cells. 1F6 human melanoma cells were stably transfected with constructs encoding either the 18 kDa (18kD) or all (ALL) bFGF isoform proteins. We observed highly increased CD13 mRNA and protein expression in the 1F6 clones regardless of the overexpression of either the 18kD or all isoform proteins. Neutral aminopeptidase activity was increased five-fold and could be inhibited by bestatin and the CD13-neutralising antibody WM15. The enhanced invasion through Matrigel, but not migration in a wound assay, was efficiently abrogated by both bestatin and WM15. Upregulation of CD13 expression was the result of increased epithelial and myeloid promoter activity up to 4.5-fold in 1F6-18kD and 1F6-ALL clones. Interestingly, in a panel of human melanoma cell lines, a significant correlation (*r*^*2*^=0.883, *P*<0.05) between bFGF and CD13 mRNA and protein expression was detected. High bFGF and CD13 expression were clearly related with an aggressive phenotype. Taken together, our data indicate that high bFGF expression upregulates CD13 expression in human melanoma cells by activating both the myeloid and the epithelial CD13 promoter. In addition, we show that high bFGF and CD13 expression results in enhanced invasive capacity and metastatic behaviour of human melanoma cells.

Cutaneous melanoma is a type of skin cancer with high metastatic potential. Melanoma cells can express a wide variety of (angiogenic) growth factors, such as basic fibroblast growth factor (bFGF), vascular endothelial growth factor (VEGF), platelet-derived growth factor (PDGF) and interleukin-8 (IL-8). During tumour development, melanoma cells depend on the autocrine production of these growth factors for proliferation and survival (Rodeck, 1993; Halaban, 1996) Most specifically, bFGF appears to be a key signal in melanoma progression. Basic fibroblast growth factor is involved in proliferation, migration and invasion of tumour cells (Bikfalvi *et al*, 1997) We here report that bFGF overexpression in human melanoma cells is responsible for upregulation of CD13 resulting in a highly increased invasive capacity.

CD13/Aminopeptidase N (CD13) is a Zn^2+^-dependent type 2 transmembrane ectopeptidase of 150 kDa that forms a noncovalently bound homodimer on the cellular membrane. Although CD13 was first described to be a marker for haematopoietic cells of myeloid origin, expression has been reported on non-haematopoietic cells and tissues, such as fibroblasts, brain cells, epithelial cells of the liver, kidney and intestine. The C-terminal part of the protein is located extracellularly and contains the active site (Riemann *et al*, 1999). CD13 preferentially cleaves neutral amino acids from small peptides rather than from large proteins. Because of its broad substrate specificity, the functional role of CD13 strongly depends on the location of expression. Previous studies have indicated that in the intestine, CD13 is involved in the terminal degradation of small peptides and amino-acid scavenging (Turner, 1986). In synaptic membranes in the brain, CD13 has been shown to inactivate endorphins and enkephalins (Matsas *et al*, 1985). In addition, CD13 participates in the processing of peptides presented by the major histocompatibility complex class II molecule (Hansen *et al*, 1993; Larsen *et al*, 1996). Furthermore, CD13 acts as the receptor for the transmissible gastroenteritis virus and the human coronavirus (Delmas *et al*, 1992).

High expression of CD13 can be detected in a number of human solid tumours, including melanoma (Menrad *et al*, 1993; Saiki *et al*, 1993; Fujii *et al*, 1995; Ishii *et al*, 2001; Hashida *et al*, 2002; Ikeda *et al*, 2003; Kehlen *et al*, 2003; Van Hensbergen *et al*, 2004), and a soluble form of CD13 has been shown to be highly increased in intratumoral fluids and ascites (Van Hensbergen *et al*, 2002). Few observations have been done with regard to the functional role of CD13 in malignancies. In melanoma cells, CD13 was reported to act as an auxiliary adhesion molecule that translocates to sites of cell–cell contact (Menrad *et al*, 1993). In renal cell carcinoma, fibrosarcoma and melanoma cell lines, CD13 facilitates invasion by degradation of the extracellular matrix (ECM) (Menrad *et al*, 1993; Saiki *et al*, 1993; Fujii *et al*, 1995). CD13 is also involved in tumour angiogenesis, since CD13 is present on tumour endothelial cells, but not on existing blood vessels in normal tissues (Pasqualini *et al*, 2000).

Menrad *et al* (1993) have been the first to describe the presence of CD13 on melanoma cells, whereas CD13 expression could not be detected on normal melanocytes. Moreover, CD13 expression becomes increasingly prevalent during the process of melanoma progression (Elder *et al*, 1989; Menrad *et al*, 1993) and is directly associated with ECM degradation and invasion. Transfection of CD13 into A375M and A2058 human melanoma cells resulted in increased degradation of type IV collagen and invasion in ECM. It was suggested that CD13 may serve as an activator of type IV collagenase required for the invasive and metastatic phenotype of melanoma cells (Fujii *et al*, 1995).

The expression of CD13 is regulated by two distinct cell type-specific promoters, the proximal or epithelial promoter and the distal or myeloid promoter. The epithelial promoter is mainly active in epithelial cells of the intestine, kidney, liver and brain and in endothelial cells, while the myeloid promoter is active in monocytes, myeloid leukaemia cells and fibroblasts (Shapiro *et al*, 1991). The two promoters are regulated by different transcription factors, but both transcripts result in expression of the same protein. In addition, both promoters are thought to be activated in a mutually exclusive manner (Shapiro *et al*, 1991). In endothelial cells, CD13 mRNA and protein expression can be transcriptionally upregulated in response to diverse factors influencing the tumour microenvironment, such as hypoxia and increased presence of angiogenic growth factors such as bFGF, VEGF, tumour necrosis factor *α* (TNF*α*) and insulin-like growth factor-1 (IGF-1) (Bhagwat *et al*, 2001). The upregulation of CD13 in endothelial cells was shown to be mediated through activation of the proximal promoter of CD13, while its regulation in melanoma cells remains to be determined.

We recently observed that overexpression of bFGF in human 1F6 melanoma cells resulted in an accelerated *in vitro* and *in vivo* growth rate. 1F6 clones displayed a different morphology, suggesting changes in the expression of adhesion molecules (Fontijn *et al*, unpublished data). Since CD13 has been implicated in melanoma invasion, we determined whether this protein was upregulated in our bFGF-overexpressing 1F6 clones. We here report that bFGF induces the expression of CD13 in human melanoma cells resulting in a highly increased invasive capacity. In addition, we report that both CD13 promoters are activated upon upregulation of CD13 by bFGF. Furthermore, we found a strong correlation between bFGF and CD13 expression in a panel of human melanoma cell lines.

## MATERIALS AND METHODS

### Cell culture

The human melanoma cell lines BLM, Mel57, M14 and 1F6 were a kind gift of Dr JR Westphal and Dr W Leenders, University Medical Centre, St Radboud Nijmegen, Netherlands, and have been established from surgically removed melanoma metastases (Van Muijen *et al*, 1991). The BRO cell line has been derived from a highly malignant and aggressive primary melanoma (Lockshin *et al*, 1985). Hep3B human hepatocellular carcinoma cells, HL60 human promyelocytic leukaemia cells and U937 human histiocytic lymphoma cells were obtained from American Type Culture Collection (ATCC, Manassa, VA, USA). Cell lines and 1F6 clones highly (H) or lowly (L) overexpressing the 18kD isoform of human bFGF (1F6-18kD-H and 1F6-18kD-L), all isoforms of bFGF (1F6-ALL-H and 1F6-ALL-L) or control empty vector cells (1F6-pcDNA3) have been described (Fontijn *et al*, unpublished data). Cells were cultured in Dulbecco's modified Eagle's medium (DMEM; Bio Whittaker, Verviers, Belgium) supplemented with 10% heat-inactivated fetal calf serum (FCS; Gibco Invitrogen, Breda, the Netherlands), 50 U ml^−1^ penicillin (ICN Biochemicals, Zoetermeer, the Netherlands) and 50 *μ*g ml^−1^ streptomycin (ICN Biochemicals). In addition, bFGF-overexpressing 1F6 clones were cultured in the presence of 500 *μ*g ml^−1^ geneticin (Gibco/Invitrogen) to maintain selection for bFGF overexpression. All cells were grown at 37°C in humidified air containing 5% CO_2_.

### Isolation of RNA and RT–PCR

Total RNA of cell lines was isolated using Trizol reagent (Gibco/Invitrogen) according to the manufacturer's protocol. RNA (3 *μ*g) was reverse-transcribed with 50 U MMLV reverse transcriptase (Invitrogen) in the presence of 1.2 *μ*l 0.5 *μ*g ml^−1^ random primers.

Quantitative Light Cycler RT–PCR was used to determine bFGF and CD13 mRNA in melanoma cells and bFGF-overexpressing 1F6 clones (Van Hensbergen *et al*, 2003) mRNA was analysed with the LightCycler-Faststart DNA Master Hybridization Probes kSYBR Green 1 kit (Roche Diagnostics, Mannheim, Germany). The human bFGF (sense: 5′-TGTGCTAACCGTTACCTGGC-3′, antisense: 5′-ATAGCTTTCCCAGGTCC-3′), CD13 (sense: 5′-GTAATACGACTCACTATAGGGCCAGGGGCCTGTACGTTTTTA-3′, antisense: 5′-AATTAACCCTCACTAAAGGGCCACCAGCTCAGTCTTGTCA-3′) and, as internal controls, human *β*2-microglobulin (sense: 5′-GATGAGTATGCCTGCCGTGTG-3′, antisense: 5′-CAATCCAAATGCGGCATCT-3′), GAPDH (sense: 5′-ACCACAGTCCATGCCATCAC-3′, antisense: 5′-TCCACCACCCTGTTGCTGTA-3′) and PDGB (sense: 5′-TCCAAGCGGAGCCATGTCTG-3′, antisense: AGAATCTTGTCCCCTGTGGTGGA-3′) genes were amplified according to the manufacturer's protocol. In short, the reaction mix contained 4 mM MgCl_2_, 0.5 *μ*M sense and antisense primer, 1 × FastStart DNA Master SYBR Green 1 mix (containing LightCycler-Faststart Enzyme, FastStart *Taq* DNA polymerase, SYBR green dye, dNTPs and reaction buffer) and 2 *μ*l cDNA. Polymerase chain reaction conditions were as follows: 95°C for 10 min followed by 40 cycles at 95°C for 10 s, 60°C for 10 s and 72°C for 22 s. cDNA was replaced by PCR-grade water as a negative control. Relative expression levels of different samples were calculated from bFGF crossing points normalised to *β*2-microglobulin. Relative mRNA expression was calculated by: (*E*^ΔCp target gene^)/(*E*^ΔCp reference gene^), in which *E*=efficiency and ΔCp=crossing point when compared to 1F6 or 1F6-pcDNA3 cells. Primer efficiencies were determined using pooled cDNAs.

In experiments designed to investigate the effect of bFGF overexpression on CD13 mRNA stability, cells were made quiescent in serum-free medium for 24 h. Next, cells were preincubated with 1 *μ*g ml^−1^ actinomycin D (Sigma-Aldrich chemie, Zwijndrecht, the Netherlands) for 1 h in serum-free medium (time point=0 h) and, thereafter, harvested after incubation for 1, 2, 3 and 5 h with 1 *μ*g ml^−1^ actinomycin D in serum-free medium. At each time –point, CD13 mRNA levels were determined by Light Cycler RT–PCR.

Expression of fragments derived from activity of the myeloid (MP) or both the myeloid and epithelial (MP/EP) promoters of CD13 was determined by RT–PCR. In total, 2 *μ*l cDNA was subjected to 38 PCR cycles with the sense primers CD13 MP: 5′-AGTCCAGGGTCCAGGTTCCA-3′ and CD13 MP/EP: 5′-ATGGCCAAGGGCTTCTATAT-3′ and the shared antisense primer CD13 MP/EP: 5′-ACGCTTTACTTTGGTCCAA-3′. As an internal control, *β*2-microglobulin expression was detected in the same samples. Polymerase chain reaction conditions were as follows: 3 min hotstart at 94°C followed by a 30 s denaturation step at 94°C, annealing at 56°C for 30 s, elongation at 72°C for 30 s. Amplification products were separated on a 1% agarose gel containing 1 *μ*g ml^−1^ ethidium bromide (Invitrogen) and analysed in a qualitative way using a Geldoc system (Bio-rad Laboratories, Veenendaal, the Netherlands).

### Western blot

Melanoma cell lines and bFGF-overexpressing 1F6 clones were lysed in ice-cold FOS-RIPA lysis buffer (10 mM Tris-HCl, 150 mM NaCl, 0.1% SDS, 0.1% NP-40 and 0.1% sodium deoxycholate) supplemented with 0.5 mM trypsin inhibitor (Sigma-Aldrich), 0.5 *μ*g ml^−1^ leupeptin (Sigma-Aldrich), 1 mM PMSF (Merck, Amsterdam, the Netherlands), 0.1 mM sodium ortho vanadate (Sigma-Aldrich) and 50 mM sodium fluoride (Baker Chemicals, Deventer, the Netherlands) for 10 min on ice. Lysates were centrifuged for 15 min at 13 000 r.p.m. at 4°C to remove debris. Protein concentrations of cell lysates were measured according to Bradford (1976).

Proteins were denatured by addition of sample buffer containing *β*-mercapto ethanol and incubation at 95°C for 5 min. Proteins were subjected to SDS–PAGE on a 12% polyacrylamide gel and electrophoretically transferred to a polyvinylidene difluoride membrane (Immobilon; Millipore, Etten-Leur, the Netherlands). Membranes were blocked for 1 h in TBST (10 mM Tris pH 8, 150 mM l^−1^ NaCl and 0.025% Tween 20)/5% milk and incubated overnight with 0.2 *μ*g ml^−1^ rabbit polyclonal human bFGF-directed antiserum (sc-79; Santa Cruz Biotechnology, Heerhugowaard, the Netherlands) or 0.5 *μ*g ml^−1^ mouse polyclonal human CD13-directed antiserum (3D8; Santa Cruz Biotechnology) in TBST/5% milk. After washing with TBST, bFGF and CD13 membranes were incubated for 1 h at room temperature with, respectively, horseradish peroxidase-conjugated mouse anti-rabbit IgG (1 : 2000 dilution; Cell Signalling Technology, Heerhugowaard, the Netherlands) or with horseradish peroxidase-conjugated rabbit anti-mouse IgG (dilution 1 : 500; DAKO Cytomation, Heverlee, the Netherlands) in TBST/5% milk. Membranes were washed and proteins were visualized by electro-chemiluminescence.

### Fluorescence-activated cell sorter analysis

Cells at 70% confluence were harvested by short trypsinisation, washed and resuspended in phosphate-buffered saline (PBS) containing 0.1% bovine serum albumin (BSA) and 0.05% NaNO_3_. 1 × 10^5^ cells were incubated at room temperature for 30 min with 1 : 20 diluted phycoerythrin (PE)-conjugated anti-human CD13 monoclonal antibody L-138 (BD biosciences, Alphen aanden Rijn, the Netherlands), PE-conjugated mouse IgG1 or PBS. Cells were washed and analysed on a FACS calibur flow cytometer (BD biosciences). Results were expressed as the ratio of the mean fluorescence of L-138 divided by that of IgG1.

### Aminopeptidase activity assay

Surface aminopeptidase activity was measured by plating 5000 cells in 10% serum in a 96-well plate according to Van Hensbergen *et al* (2002). After a 6-h incubation period, medium was removed and fresh serum-free medium containing 0.1% BSA was added. After 16 h, cells were washed with PBS and incubated with 100 *μ*l alanine-AMC (L-alanine-4-methyl-7-coumarinylamide trifluoroacetate; Sigma-Aldrich) in the absence or presence of 350 *μ*M bestatin (Sigma-Aldrich) or 10 *μ*g ml^−1^ CD13-neutralising mouse monoclonal antibody WM15 (BD biosciences). Release of the fluorescent product 7-AMC was monitored every 5 min during 1 h in a Spectrafluor multiplate reader (Tecan, Salzburg, Austria) with excitation wavelength *λ*^360^ nm and emission wavelength *λ*^465^ nm. The neutral aminopeptidase activity was calculated from the slope of the fluorescence–time curve, using a calibration curve of 7-AMC (Sigma-Aldrich).

### Migration assay

Cells were seeded in 24-well plates and grown to confluence. A wound was scratched in the confluent cell layer with a sterile pipette tip in two directions. Medium was replaced by medium without or with 10 *μ*g ml^−1^ mouse IgG, 10 *μ*g ml^−1^ WM15 or 350 *μ*M bestatin. Immediately after wounding and at time points 6, 12 and 24 h, wounds were photographed at × 25 magnification with a confocal laserscan microscope (Leica TCS 4D; Leica, Jena, Germany) and a video camera attached to a computer with Leica Q500MC software (Leica). The width of the wounds was measured in four areas at all time points and compared to the original wound at time point 0 h. Possible toxicity of IgG, WM15 and bestatin was determined by performing a parallel MTT (3-(4,5-dimetylthiazol-2-yl)-2,6-dimethyl-morpholino)-2,5-diphenyl-tetrazolium bromide; Sigma-Aldrich) assay (Mosmann, 1983) using four replicate wells and an exposure time of 24 h. Cell growth of untreated cells was set at 100% and growth of treated cells was expressed as a percentage of control cell growth.

### Matrigel invasion assay

The Matrigel invasion assay was performed in a 24-well plate transwell system (Van Hensbergen *et al*, 2003). Filters with 8 *μ*m pore size (HTS Fluoroblok Insert; Becton Dickinson labware, Le Pont de Claix, France) were coated on the lower side with 1% fibronectin (Merck) for 2 h and washed with PBS before coating the upper side of the filter with Matrigel (5 or 20 *μ*g filter^−1^; Sigma-Aldrich). DMEM containing 10% FCS was added to the lower compartment. 2 × 10^5^ cells in DMEM containing 0.5% FCS in a volume of 200 *μ*l were added to the upper compartment. In control wells, 0.5% FCS was added to both the lower and the upper compartment. For inhibition of invasion, 350 *μ*M bestatin was added to both the upper and the lower compartment. WM15 (10*μ*g ml^−1^) was present in the upper compartment only, and this included a 1-h preincubation period before the cells were seeded. Cells were allowed to invade for 48 h at 37°C. Invasion was quantified by exposing the cells in the lower compartment to 5 *μ*M calcein-AM (Molecular Probes, Leiden, the Netherlands) at 37°C during the final 30 min of the assay. Calcein-AM is the acetoxy-methyl ester of calcein, which diffuses readily through the cell membrane. In the cytosol, calcein-AM is converted into the fluorochrome calcein by esterases. Calcein fluorescence in the lower compartment was measured in a Spectrafluor multiplate reader (Tecan) with excitation wavelength *λ*^492^ nm and emission wavelength *λ*^535^ nm. Cytotoxicity of WM15 and bestatin was determined by performing a parallel MTT assay with an exposure time of 48 h in a similar way as described for the migration assay.

### Transient transfection and dual luciferase assay

The CD13 promoter-luciferase reporter constructs BstXI-luciferase, encoding the epithelial promoter and CD13-1.15-luciferase, encoding the myeloid promoter, were a kind gift of Dr LH Shapiro and have been described previously (Bhagwat *et al*, 2001). pGL2 basic-luciferase and pRenilla luciferase-CMV were purchased from Promega (Leiden, the Netherlands). Exponentially growing 1F6 cells and bFGF-overexpressing 1F6 clones in duplicate wells of six-well plates were transiently transfected with 0.5 *μ*g of each reporter plasmid for 48 h using Fugene 6 transfection reagent (Roche Diagnostics) following the manufacturer's protocol. Cotransfection with 5 ng pRenilla luciferase-CMV (pRL-CMV) was performed to correct for possible differences in transfection efficiency. The activities of firefly and Renilla luciferase were determined with the dual luciferase reporter assay (Promega) following the manufacturer's protocol. In short, cells were lysed by scraping in the presence of 250 *μ*l 1 × passive lysis buffer. In total, 20 *μ*l of cell lysate was transferred into the luminometer tube containing 100 ml LAR II buffer (Promega), and firefly luciferase activity was measured. Consecutively, Renilla luciferase activity was measured after adding 100 ml of Stop & Glo Reagent (Promega). Relative CD13 promoter activity in 1F6 cells and bFGF-transfected clones was calculated by the formula: 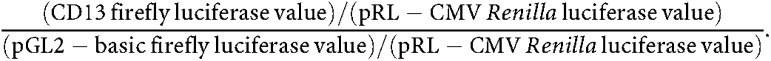


### Statistical analysis

Statistical analysis of possible differences between mean values was performed with a two-tailed Student's *t*-test. *P*<0.05 was considered significant.

## RESULTS

### Characteristics of bFGF-overexpressing 1F6 cells

Recently, we characterised 1F6 melanoma cells stably transfected with the 18kD isoform of bFGF, ALL isoforms of bFGF or a control empty vector pcDNA3 (Fontijn *et al*, unpublished data). The overexpressed bFGF was biologically active in a human umbilical vein endothelial cell (HUVEC) proliferation assay. Changes in cell morphology were observed in 1F6 cells overexpressing both the 18kD and ALL bFGF isoforms. In contrast to 1F6 cells, which are large cells with numerous dendritic cell processes, 1F6-18kD and 1F6-ALL cells appeared smaller, were more rounded and formed less dendritic cell processes. In addition, significantly accelerated *in vitro* and *in vivo* growth rates up to, respectively, two- and three-fold were observed in 1F6 cells and tumours overexpressing either 18kD or ALL bFGF isoforms (Fontijn *et al*, unpublished data). Since these changes in morphology and growth rate might be indicative for increased aggressive behaviour, we determined whether CD13 expression was upregulated in bFGF-overexpressing 1F6 cells.

### CD13 expression and activity is increased in 1F6 cells overexpressing 18kD or ALL isoforms of bFGF

**Figure 1:**
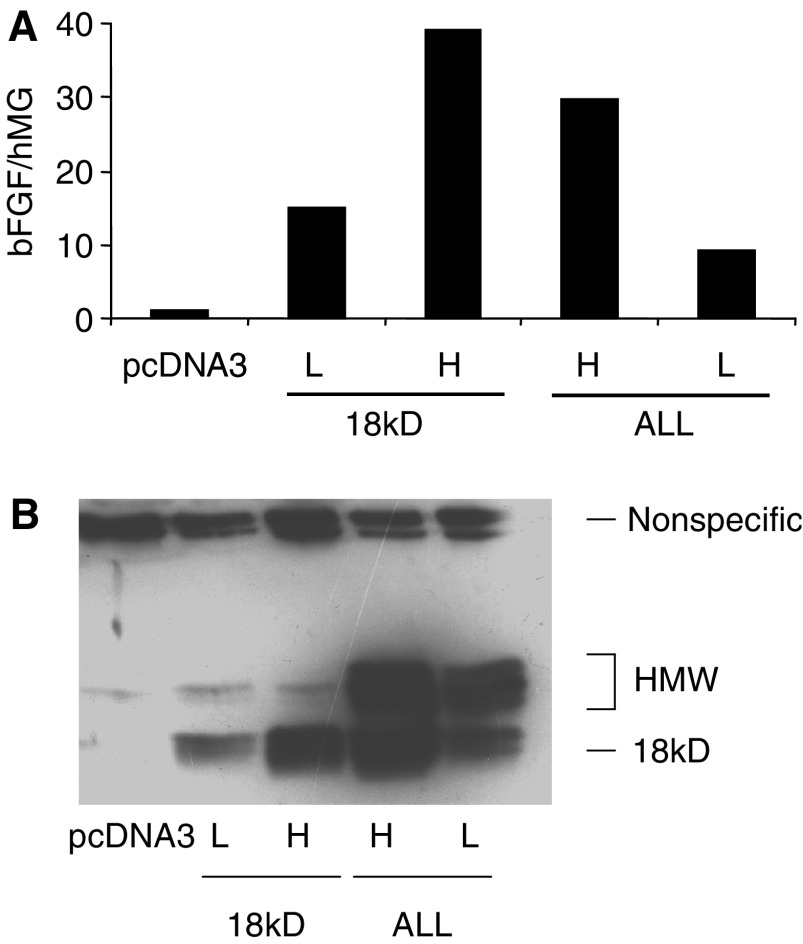
Basic fibroblast growth factor mRNA and protein expression in 1F6 cells stably transfected with 18kD or ALL isoforms of bFGF. (**A**) Mean ratios between bFGF and human *β*2-microglobulin (hMG) mRNAs in 1F6-pcDNA3 and 1F6 clones with high (H) or low (L) 18 kD or ALL bFGF isoform proteins were determined by Light Cycler RT–PCR. The bFGF/hMG ratio in 1F6-pcDNA3 cells was set at 1. (**B**) Basic fibroblast growth factor proteins from the same cell lines were visualised by subjecting 20 *μ*g protein per lane to Western blot.

**Figure 2:**
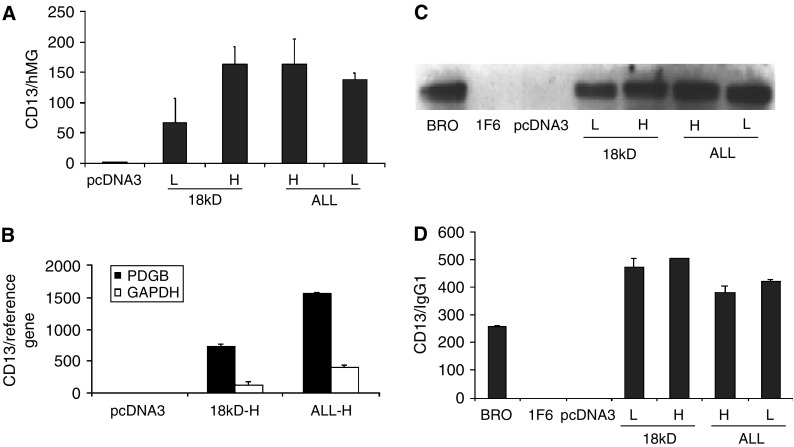
CD13 mRNA and protein expression in BRO, 1F6, 1F6-pcDNA3 and 1F6 clones with high (H) or low (L) 18kD or ALL bFGF isoform proteins. (**A**, **B**) CD13 mRNA expression was determined by Light Cycler RT–PCR. Mean relative expression ratios (±s.d.) between CD13 and hMG, GAPDH or PDGB are shown as calculated from duplicate samples. The CD13/reference gene ratio in 1F6-pcDNA3 cells was set at 1. (**C**) CD13 protein expression was determined by subjecting 50 *μ*g protein of each cell line to Western blot. (**D**) Fluorescence-activated cell sorter analysis of CD13 protein expression. Cells were incubated at room temperature with 1 : 20 diluted PE-conjugated anti-human CD13 monoclonal antibody L-138 or control mouse IgG1 for 30 min. Depicted are mean CD13/IgG1 ratios (±range) of duplicate samples. The experiments were performed at least twice with similar outcome.

bFGF mRNA and protein expression in 1F6-pcDNA3 cells and 1F6 clones lowly or highly overexpressing 18kD (18kD-L, 18kD-H) or ALL (ALL-L, ALL-H) isoforms of bFGF are visualised in Figure 1. Increased CD13 mRNA expression up to 150-fold was observed in 1F6-18kD as well as in 1F6-ALL clones as compared to the expression level in 1F6-pcDNA3 (Figure 2A). Figure 2B shows CD13 mRNA expression in the same clones, but normalised for two other reference genes GAPDH and PDGB. A similar pattern of CD13 upregulation was observed, although the absolute fold increase over 1F6-pcDNA3 CD13 expression levels varied with the reference gene of choice. GeNorm analysis (Vandesompele *et al*, 2002) indicated that among the reference genes, human *β*2-microglobulin and GAPDH were the most stable in our model (data not shown). High expression of CD13 in bFGF-transfected 1F6 clones was confirmed on the protein level by Western blot (Figure 2C). Fluorescence-activated cell sorter analysis also confirmed high CD13 protein expression, which was increased up to 450-fold in bFGF-overexpressing 1F6 clones (Figure 2D). CD13 expression in 1F6 clones was up to two-fold higher than that in the BRO cell line, which contains high levels of endogenous CD13.

**Figure 3:**
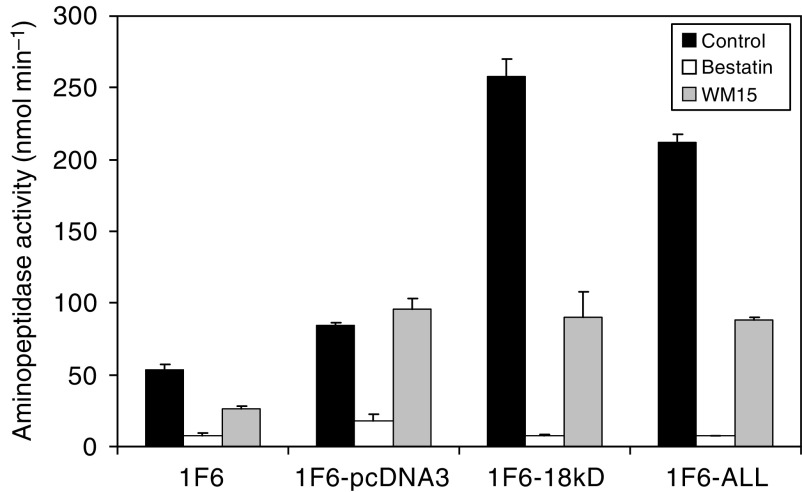
Neutral aminopeptidase activity in 1F6, 1F6-pcDNA3 and 1F6 clones highly overexpressing 18kD or ALL bFGF isoform proteins. Aminopeptidase activity was determined in the absence or presence of 350 *μ*M bestatin or 10 *μ*g ml^−1^ WM15 from the slope of the fluorescence–time curve, using a calibration curve of 7-AMC, and was expressed as nmol min^−1^. Mean values (±s.d.) are shown of triplicate samples.

To test whether the overexpressed CD13 had enzymatic activity, aminopeptidase-activated release of 7-AMC from alanine-AMC was measured in 1F6, 1F6-pcDNA3 and clones highly overexpressing 18kD or ALL bFGF isoform proteins (Figure 3). In line with mRNA and protein expression, aminopeptidase activity was increased up to five-fold in bFGF-overexpressing clones. Addition of 350 *μ*M bestatin, a nonspecific aminopeptidase inhibitor, resulted in a more than 90% inhibition of enzyme activity in all cell lines (*P*<0.05). In contrast, the specific CD13-neutralising antibody WM15 (10 *μ*g ml^−1^) did not inhibit the enzymatic activity in 1F6 and 1F6-pcDNA3 cells, suggesting that CD13 is not involved in the basal neutral aminopeptidase activity. In 1F6-18kD and 1F6-ALL cells, however, exposure to WM15 resulted in a three-fold reduction of total aminopeptidase activity, indicating that the increased enzymatic activity in 1F6-18kD and 1F6-ALL cells is mainly due to increased CD13 levels.

### High CD13 expression in bFGF-overexpressing 1F6 clones facilitates invasion, but not migration

**Figure 4:**
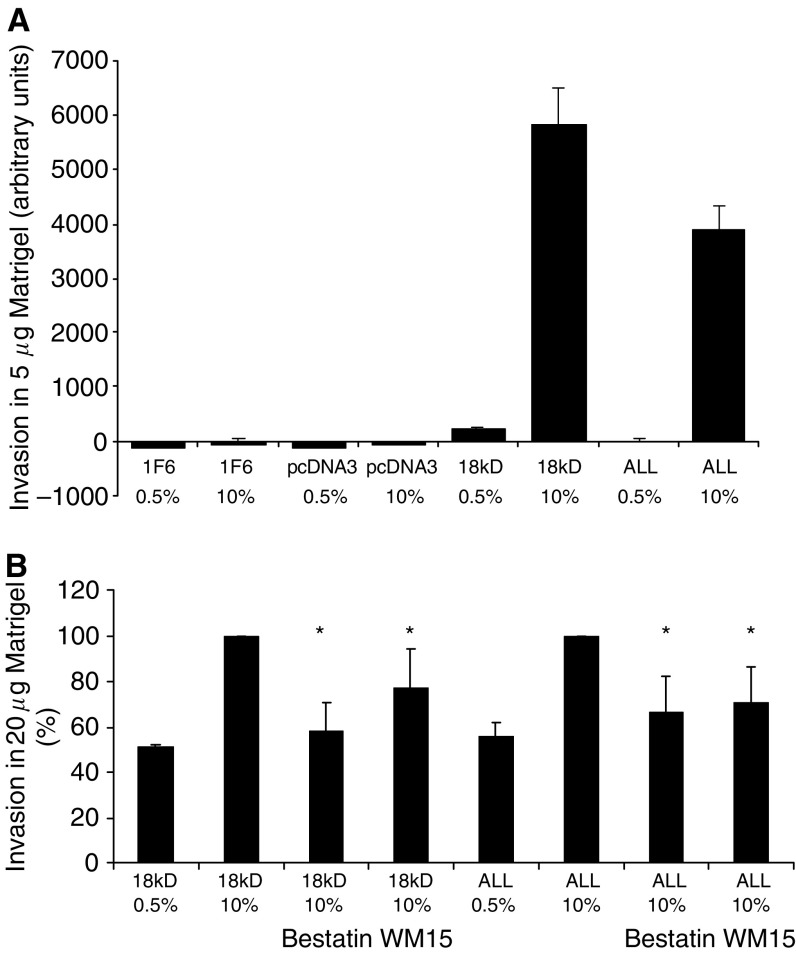
Invasion of 1F6, 1F6-pcDNA3 and 1F6 clones highly overexpressing 18kD or ALL bFGF isoform proteins in Matrigel. In the upper compartment, 0.2 × 10^6^ cells were added in 0.5% FCS-containing medium. Spontaneous invasion was determined by medium containing 0.5% FCS in the lower compartment, while 10% FCS was used as a chemoattractant in the lower compartment to induce invasion. The assay was terminated at 48 h. (**A**) Invasion of cells through Matrigel at 5 *μ*g filter^−1^ under 0.5 or 10% serum conditions. (**B**) Inhibition of 1F6-18kD and 1F6-ALL invasion through Matrigel at 20 *μ*g filter^−1^ by addition of 350 *μ*M bestatin and 10 *μ*g ml^−1^ WM15. The 10% FCS-induced invasion of clones 1F6-18kD and 1F6-ALL was set at 100%. Mean values (±s.d.) of three independent experiments carried out in duplicate wells are shown. ^*^Indicates a significant difference (*P*<0.05) as compared to 10% FCS-induced invasion.

CD13 has been implicated to be required for melanoma cell invasion in ECM *in vitro* as well as *in vivo* (Menrad *et al*, 1993; Fujii *et al*, 1995). Therefore, we determined whether the increased CD13 expression observed in the highly bFGF-overexpressing 1F6 clones resulted in a higher invasive capacity. In contrast to 1F6 and 1F6-pcDNA3 cells, which did not show any invasion through Matrigel 5 *μ*g filter^−1^ in the presence of 10% FCS used as a chemoattractant, highly increased invasion was observed for both 1F6-18kD and 1F6-ALL clones (Figure 4A). Figure 4B shows that 1F6-18kD and 1F6-ALL clones even invaded through Matrigel 20 *μ*g filter^−1^. Addition of 350 *μ*M bestatin resulted in complete inhibition of the FCS-induced invasion (*P*<0.05). Exposure to 10 *μ*g ml^−1^ of the CD13-neutralising antibody WM15 also resulted in a significant reduction of invasion (*P*<0.05). Toxicity of bestatin and WM15 was determined by an MTT assay. Bestatin at 350 *μ*M resulted in a growth inhibition of approximately 10%, while WM15 at 10 *μ*g ml^−1^ did not affect cell growth (data not shown).

**Figure 5:**
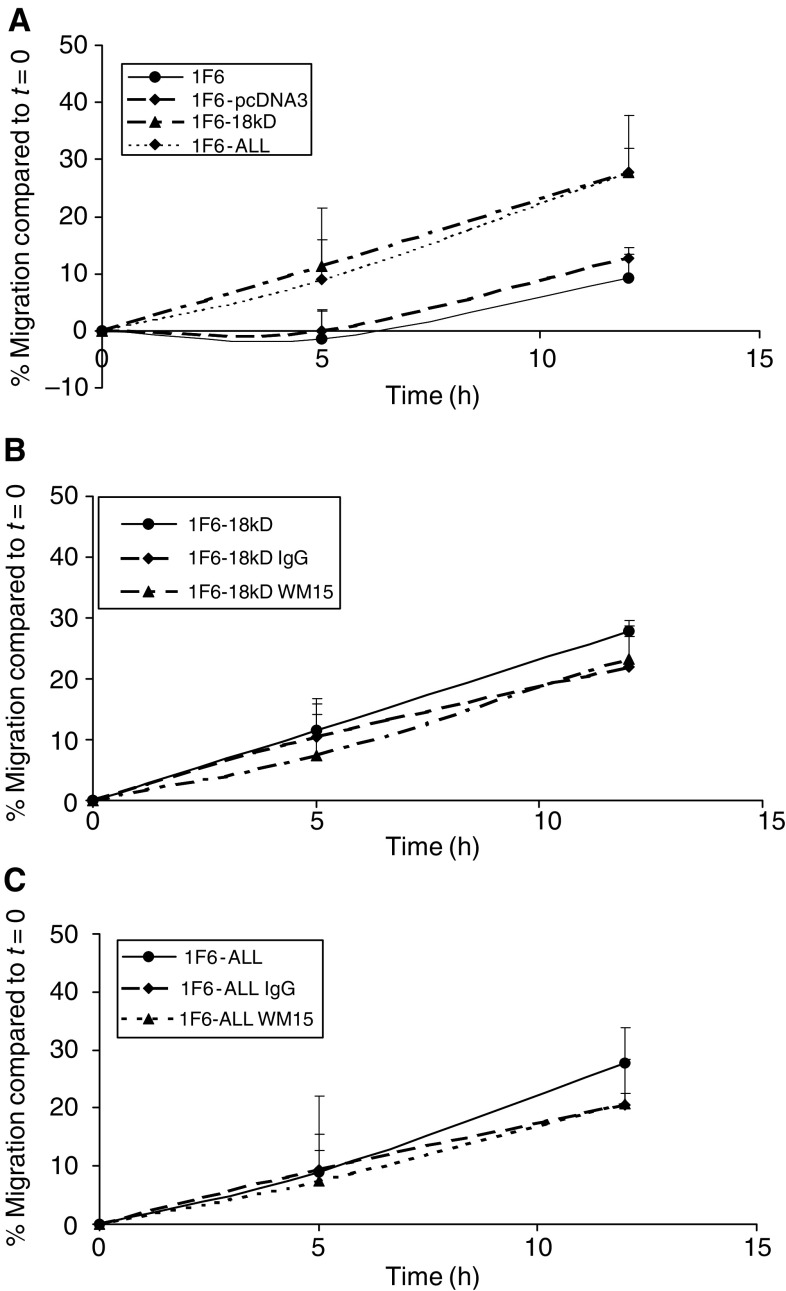
Scratch wound assay for 1F6, 1F6-pcDNA3 and 1F6 clones highly overexpressing 18kD or ALL bFGF isoform proteins. Migration of cells in wounds at time points *t*=5 h and *t*=12 h was expressed as a percentage of the width of the wound at *t*=0. (**A**) Spontaneous migration of 1F6, 1F6-pcDNA3, 1F6-18kD and 1F6-ALL cells. (**B**) Migration of 1F6-18kD cells in the absence or the continuous presence of mouse IgG1 (control antibody) or WM15. (**C**) Migration of 1F6-ALL cells in the absence or the continuous presence of mouse IgG1 (control antibody) or WM15. Mean values (±s.d.) are given of eight observations.

The migratory capacity of 1F6 cells and bFGF-overexpressing clones was determined by a wound assay. Figure 5A illustrates that clones 1F6-18kD and 1F6-ALL clearly had a higher rate of migration as compared to that of parent 1F6 and 1F6-pcDNA3 cells (*P*<0.05). Exposure to 10 *μ*g ml^−1^ WM15 did not inhibit the migration rate (Figure 5B and C) indicating that CD13 activity is not involved in this process.

### Basic fibroblast growth factor-induced CD13 expression is not due to increased mRNA stability

**Figure 6:**
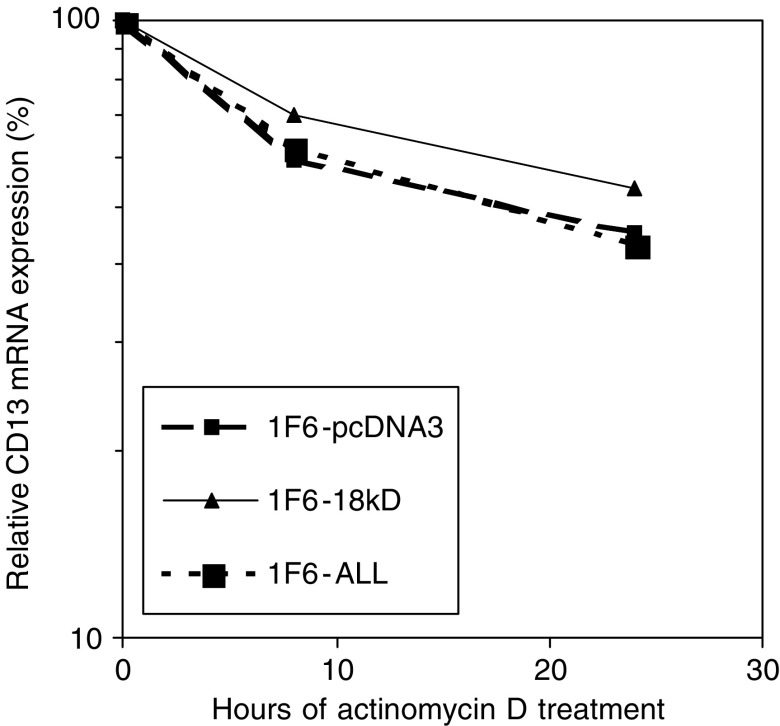
Determination of CD13 mRNA half-life in 1F6-pcDNA3 and 1F6 clones highly overexpressing 18kD or ALL bFGF isoform proteins. Cells were treated with the transcription inhibitor actinomycin D for the time periods specified. The CD13/*β*2-microglobulin mRNA ratio was determined by Light Cycler RT-PCR and expressed as a percentage of the ratio at the start of the experiment.

One possible mechanism for the increased CD13 expression observed in bFGF-overexpressing 1F6 cells is an increase in CD13 mRNA stability. To test this possibility, we determined the degradation of CD13 mRNA in 1F6-pcDNA3 and bFGF-transfected 1F6 clones after incubation with the transcription inhibitor actinomycin D for different time periods by Light-Cycler PCR. From Figure 6 it can be seen that CD13 mRNA in 1F6-pcDNA3, 1F6-18kD and 1F6-ALL cells was degraded with similar half-lifes of approximately 18 h.

### Basic fibroblast growth factor can induce expression of CD13 transcripts from the myeloid promoter

**Figure 7:**
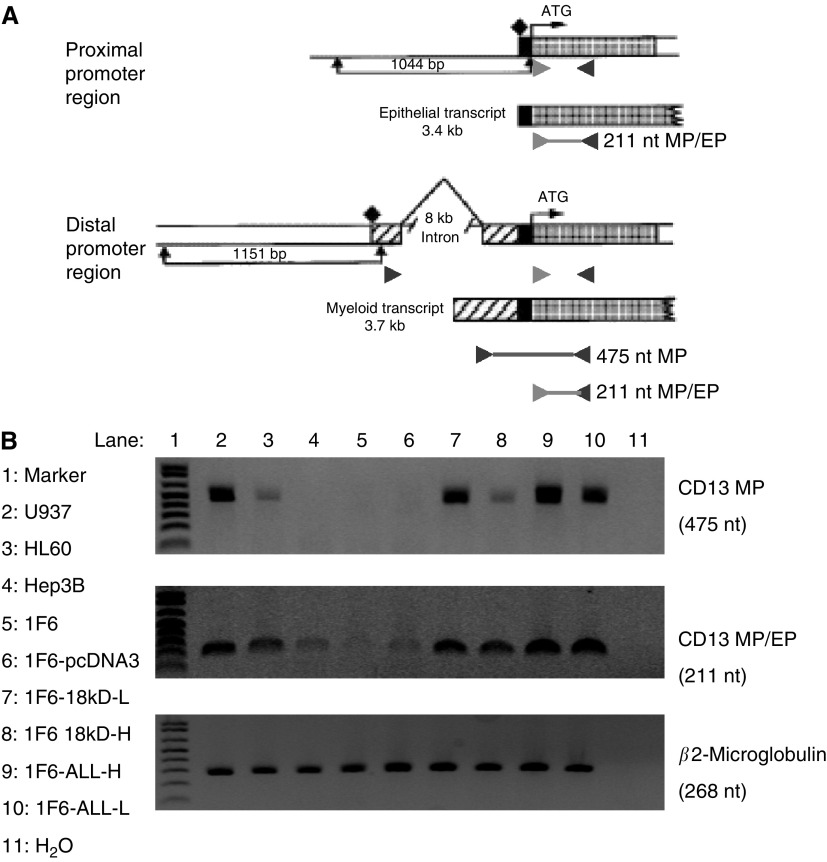
RT–PCR analysis for determination of CD13 expression from the myeloid and epithelial promoter in 1F6, 1F6-pcDNA3 and 1F6 clones with high (H) or low (L) 18kD or ALL bFGF isoform proteins. (**A**) Design of primer sets for specific amplification of fragments derived from the myeloid promoter (CD13 MP) and for expression of CD13 from both the myeloid and epithelial promoter (CD13 MP/EP) (see Materials and Methods). (**B**) Amplification of CD13 MP and CD13 MP/EP promoter fragments in 1F6 cells and bFGF-overexpressing clones. U397 and HL60 cells were included as positive controls for expression of myeloid CD13 promotor fragments and Hep3B cells as a negative control for myeloid promoter expression. Human *β*2-microglobulin was used as an internal control.

CD13 transcriptional regulation can be mediated by the distal, myeloid promoter, and a proximal, epithelial promoter (Shapiro *et al*, 1991). We designed a primer set to specifically detect CD13 mRNA expression resulting from activity of the myeloid promoter (CD13 MP). U937 and HL60 cells were included as positive controls for myeloid CD13 promoter activity. As a negative control, Hep3B cells were used, since CD13 expression in this cell line is the result of activity of the epithelial promoter only. While no myeloid promoter fragment could be detected in Hep3B, 1F6 and 1F6-pcDNA3 cells (Figure 7, lanes 4–6), high levels of myeloid promoter fragments were observed in 1F6 clones overexpressing 18kD and ALL bFGF isoforms (Figure 7, lanes 7–10). A second primer set (CD13 MP/EP) was used to amplify fragments resulting from activity of both the epithelial and the myeloid promoter. As expected, positive bands were observed in all cell lines using this primer set.

### Increased CD13 expression is mediated by enhanced activity of both the myeloid and epithelial promoter

**Figure 8:**
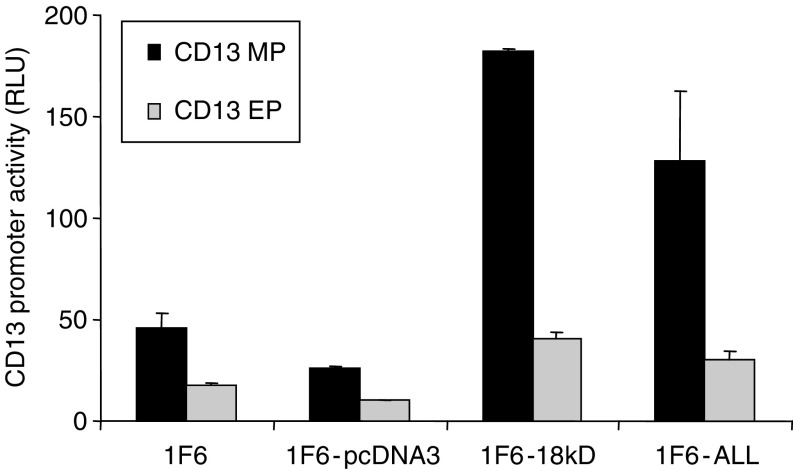
CD13 myeloid and epithelial promoter activity in 1F6, 1F6-pcDNA3 and 1F6 clones highly overexpressing 18kD or ALL bFGF isoform proteins. Luciferase-coupled CD13 myeloid (MP) or epithelial (EP) promoter construct (1 *μ*g) and pGL2-basic vector were transiently transfected using a *μ*g DNA : *μ*l Fugene ratio of 3 : 1. Cotransfection with 5 ng pRL-CMV was performed to correct for differences in transfection efficiency between cell lines. Mean values of relative light units (RLU±range) of duplicate samples are shown. The experiment was repeated three times with similar outcome.

Our RT–PCR analysis indicated that at least the CD13 myeloid promoter is involved in the increased CD13 expression observed in bFGF-overexpressing 1F6 clones. To further examine whether only the myeloid or both the myeloid and epithelial CD13 promoters were activated, transient transfections were performed using reporter gene constructs encoding for either the myeloid promoter (BstXI-luc) or the epithelial promoter (CD13-1.15-luc). To correct for possible differences in transfection efficiency between parent 1F6 cells and bFGF-overexpressing clones, cotransfections were performed with pRenilla-CMV. CD13 myeloid promoter activity in clones 1F6-18kD-H and 1F6-ALL-H was, respectively, 4.5- and 2.8-fold induced. Interestingly, the activity of the epithelial promoter was also induced up to 2.5-fold in both bFGF-overexpressing clones (Figure 8). These data clearly indicate that, although basal activity of the myeloid promoter in 1F6 cells was about 2.5-fold higher as compared to the activity of the epithelial promoter, both CD13 promoters are activated in bFGF-overexpressing 1F6 cells.

### CD13 expression correlates with bFGF expression in a panel of human melanoma cell lines

**Figure 9:**
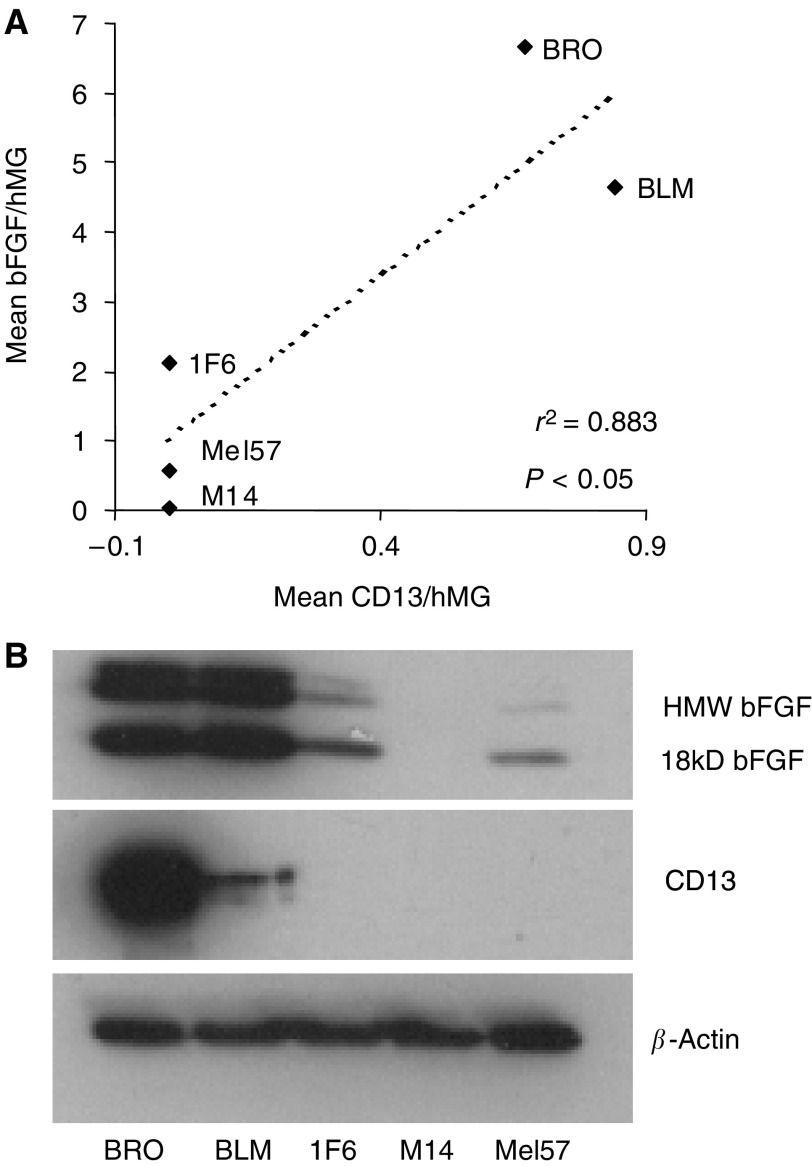
(**A**) Correlation between bFGF and CD13 mRNA expression in a panel of human melanoma cell lines. cDNAs were analysed for bFGF and CD13 mRNA expression with Light Cycler RT–PCR and plotted as mean relative expression ratios between bFGF, CD13 and human *β*2-microglobulin (hMG) of two independent experiments. Significance was determined with the Spearman's correlation test. (**B**) Basic fibroblast growth factor and CD13 protein expression in a panel of human melanoma cell lines. Of each cell line, 20 *μ*g (bFGF) or 50 *μ*g (CD13) protein of total cell lysates was subjected to Western blot. Blots were stripped and stained with anti-*β*-actin antibody to confirm equal protein loading.

Since we observed that bFGF overexpression can induce CD13 expression in human melanoma cells, we determined whether there was a correlation between bFGF and CD13 mRNA expression in a panel of melanoma cell lines. Indeed, the two cell lines, BRO and BLM, with high bFGF expression also contained a high level of CD13 mRNA, while low CD13 mRNA content was present in Mel57, M14 and 1F6 cells expressing low levels of bFGF mRNA (Figure 9A). A significantly positive correlation was calculated between CD13 and bFGF mRNA expression (*P*< 0.05, *R*^*2*^=0.883). In accordance with mRNA data, CD13 protein expression was only observed in BRO and BLM cell lines containing high levels of bFGF proteins (Figure 9B).

## DISCUSSION

We report for the first time that overexpression of bFGF in human melanoma cells mediates highly increased expression of CD13 mRNA and protein, which indicates that bFGF is involved in the induction of an invasive cellular phenotype. The increased invasiveness of bFGF-overexpressing clones through Matrigel could be attributed to high CD13 expression, since the specific monoclonal antibody WM15 inhibited this process. In accordance with these effects of bFGF overexpression, we found in a panel of human melanoma cell lines a highly significant correlation between endogenous bFGF and CD13 mRNA expression as well as spontaneous metastasis formation. These findings point towards an important role for bFGF in the high metastatic potential of melanomas in patients.

For our experiments, we made use of 1F6 cells with low endogenous bFGF levels that were transfected with vectors either encoding the 18kD isoform or ALL isoforms in an attempt to distinguish between the variation in phenotypic behaviour induced by the different proteins. The 18kD bFGF isoform functions as an autocrine and paracrine growth factor and is mainly found in the cytoplasm of cells and bound to heparan sulphate proteoglycans (HSPGs) on the cell membrane. In contrast, the high molecular weight (HMW) isoforms are located in the nucleus and are thought to play a role in transcriptional regulation (Renko *et al*, 1990; Bugler *et al*, 1991; Florkiewicz *et al*, 1991; Quarto *et al*, 1991). Upon transfection, however, both 1F6-18kD and 1F6-ALL clones expressed similar amounts of CD13, which suggests that expression of the 18kD protein alone is sufficient for the induction of CD13. Moreover, changes in morphology and growth rate were similar between 1F6-18kD and 1F6-ALL clones.

Induction of CD13 expression by exogenous bFGF has previously been described in human endothelial cells and is required for vasculogenesis. In the study by Bhagwat *et al* (2001), addition of the 18kD bFGF protein to serum-starved HUVECs upregulated CD13 mRNA expression up to 2.5-fold. Although bFGF was the most potent stimulator of CD13 expression in that study, induction of CD13 protein was also observed when endothelial cells were cultured under hypoxic conditions or were treated with the angiogenic growth factors VEGF, TNF*α* or IGF-1. Inhibition of CD13 expression by bestatin, amastatin or the monoclonal-anti CD13 antibody MY7 resulted in a clear reduction of capillary tube formation of HUVECs, while there was no effect of these inhibitors on proliferation (Bhagwat *et al*, 2001). The authors have also reported that exogenous bFGF could induce CD13 expression in KS1767 Kaposi sarcoma cells (Bhagwat *et al*, 2001). Recently, Kehlen *et al* (2003) have shown induction of CD13 expression upon stimulation of serum-starved 1736 thyroid carcinoma cells with bFGF.

Repeatedly, we were not able to increase CD13 expression by stimulating serum-starved 1F6 cells with exogenous recombinant human bFGF, regardless efficient stimulation of cell proliferation (data not shown). This finding indicates that the bFGF-mediated induction of CD13 in 1F6 cells is an indirect effect of the transformation process in itself, rather than a direct effect on CD13 promoter activity. Since CD13 can be detected on melanoma cells (Elder *et al*, 1989; Menrad *et al*, 1993; Fujii *et al*, 1995), we are the first to report that endogenous bFGF can mediate the expression and activity of this protein.

Bestatin is a well-known chemical inhibitor of CD13 aminopeptidase activity, which blocks the catalytic site of the enzyme in a competitive manner. Although bestatin is often considered to be specific for CD13, it also inhibits other aminopeptidases, such as aminopeptidase B and leucine aminopeptidases (Scornik and Botbol, 2001). High concentrations of bestatin (150–300 *μ*M) clearly reduced invasion of HT1080 fibrosarcoma cells and WM1158 melanoma cells through Matrigel (Menrad *et al*, 1993; Fujii *et al*, 1996). WM15 is a monoclonal antibody that specifically neutralises CD13 activity. WM15 has been shown to inhibit invasion through Matrigel, as described for SN12M renal-cell carcinoma, HT1080 fibrosarcoma and A375 melanoma cells (Saiki *et al*, 1993). In our bFGF-overexpressing 1F6 clones, both bestatin and WM15 almost completely abrogated the enhanced invasive capacity. This observation indicates that the increased CD13 expression and activity in the bFGF-overexpressing clones is mainly responsible for the facilitated invasive potential.

The activity of the myeloid and the epithelial promoters of CD13 is believed to be mutually exclusive (Shapiro *et al*, 1991). As a result, expression of CD13 protein in a particular cell type is thought to be mediated by activation of either the myeloid or the epithelial promoter. We now show that both CD13 promoters can be activated in response to bFGF overexpression. Although in 1F6 cells the basal expression of the myeloid promoter was consistently found to be higher than the activity of the epithelial promoter, both activities were increased to the same extent, up to four-fold in clones 1F6-18kD and 1F6-ALL. We do not know if the current results are specific for melanoma cells, or whether the use of the more sensitive techniques would also allow to detect upregulation of both promoters in other cell types. While initial CD13 promoter studies were performed with chloramphenicol acetyl transferase (CAT) assays and Northern blots (Shapiro *et al*, 1991), we made use of RT–PCR and promoter-luciferase assays allowing us to detect the activities of both promoters in 1F6 melanoma cells and clones.

Recently, induction of CD13 expression by bFGF in endothelial cells was shown to be regulated by two Ras-mediated signalling pathways that have been implicated in the transition of quiescent to active endothelium: the mitogen-activated protein kinase (MAPK) and phosphoinositide-3 kinase/AKT (PI-3K/AKT) pathways (Bhagwat *et al*, 2003). Petrovic *et al* (2003) have demonstrated in activated endothelial cells that CD13 transcription was induced by RAS/MAPK-mediated phosphorylation of the transcription factor Ets-2 resulting in increased activity of the epithelial CD13 promoter. In our bFGF-overexpressing 1F6 clones, phospho-AKT and phospho-p38 MAPK levels were increased as compared to basal levels in parent 1F6 cells, while basal phospho-ERK1/2 MAPK was not further increased (Fontijn *et al*, unpublished data). We are further investigating the possible contribution of the PI-3K/AKT and p38 MAPK signalling pathways in the increased CD13 expression in bFGF-overexpressing 1F6 clones.

In general, high CD13 expression in solid tumours in patients is considered as an unfavourable factor. Few clinical studies are available, among which are observations in pancreatic carcinoma, colon carcinoma and thyroid carcinoma, which will be described shortly. CD13 protein expression was detected in 48% of pancreatic carcinoma patients and was significantly associated with a shorter median survival (Ikeda *et al*, 2003). The disease-free and overall survival rate for patients with CD13-positive colon carcinoma was significantly lower than that for patients without CD13 expression (Hashida *et al*, 2002). Kehlen *et al* (2003) have shown that CD13 expression in undifferentiated thyroid carcinomas was higher than that in papillary or follicular thyroid carcinomas, suggesting that it is a marker for differentiation. Clinical studies on CD13 expression in cutaneous melanoma are yet to be carried out.

It can be hypothesized that the presence of CD13 in melanoma patients will also be associated with poor prognosis. As a rationale, it has been shown *in vitro* that inhibition of CD13 aminopeptidase activity can reduce the invasive capacity of WM1158 and A375M melanoma cells through Matrigel (Menrad *et al*, 1993; Saiki *et al*, 1993). Upon transfection of CD13 into A375M and A2058 melanoma cells, increased degradation of type IV collagen and invasion in ECM have been observed (Fujii *et al*, 1995). These CD13-transfected cells showed a significantly augmented lung-colonising potential in nude mice (Fujii *et al*, 1995). The observations are in agreement with our data that 1F6 cells could hardly invade through Matrigel, but their invasive capacity is greatly facilitated in the case of CD13 overexpression. As proof of concept, in our panel of human melanoma cell lines, we found a clear correlation between bFGF and CD13 mRNA and protein expression (Figure 9). High bFGF and CD13 protein expression could be detected in melanoma cell lines BRO and BLM, being aggressive cell lines on the basis of a high *in vitro* and *in vivo* growth rate. Furthermore, BLM and BRO cells grown as subcutaneous xenografts in nude mice have a very high rate of spontaneous metastasis formation (Lockshin *et al*, 1985; Van Muijen *et al*, 1991).

Bestatin has been studied for its therapeutic usefulness in the clinic in the past, since the compound was considered to have immunopotentiating activity (Ota, 1991). In a prospective randomised trial in adult nonlymphocytic leukaemia, prolongation of remission duration and survival was achieved with bestatin added to maintenance chemotherapy in elderly patients (Ota *et al*, 1986). Prospective randomised clinical trials in various solid tumours contained either too few patients or were negative to demonstrate a beneficial effect on survival (Ota, 1991). In a recent, randomised double-blind placebo-controlled trial by Ichinose *et al* (2003), bestatin was given for its aminopeptidase inhibiting, immunostimulant and antitumour activity to patients with completely resected stage I squamous-cell lung carcinoma. Of the 402 patients that entered the study, it appeared that the overall survival and cancer-free survival were both significantly different in favour of the bestatin-treated group. Since we found a clear correlation between bFGF and CD13 expression and invasiveness of melanoma cells, the incorporation of bestatin in a clinical trial in stage II melanoma patients should be considered with the aim of preventing the development of metastases and to improve survival.

In conclusion, our data indicate that high bFGF expression in human melanoma cells is a major mechanism of the upregulation of CD13 resulting in enhanced invasive capacity and metastatic behaviour. Selective inhibition of CD13, or even of bFGF itself, should therefore be an attractive strategy for the treatment of advanced melanoma, but likely also for adjuvant treatment in stage II disease.
